# Novel predictors for livebirth delivery rate in patients with idiopathic non-obstructive azoospermia based on the clinical prediction model

**DOI:** 10.3389/fendo.2023.1233475

**Published:** 2023-10-16

**Authors:** Li Zhang, Yuan-yuan Wang, Xiao-ying Zheng, Li lei, Wen-hao Tang, Jie Qiao, Rong Li, Ping Liu

**Affiliations:** ^1^ Center for Reproductive Medicine, Department of Obstetrics and Gynecology, Peking University Third Hospital, Beijing, China; ^2^ National Clinical Research Center for Obstetrics and Gynecology, Peking University Third Hospital, Beijing, China; ^3^ Key Laboratory of Assisted Reproduction (Peking University), Ministry of Education, Beijing, China; ^4^ Beijing Key Laboratory of Reproductive Endocrinology and Assisted Reproductive Technology, Department of Obstetrics and Gynecology, Peking University Third Hospital, Beijing, China; ^5^ Department of Urology, Peking University Third Hospital, Beijing, China; ^6^ Beijing Advanced Innovation Center for Genomics, Peking University, Beijing, China; ^7^ Peking-Tsinghua Center for Life Sciences, Peking University, Beijing, China

**Keywords:** prediction model, intracytoplasmic sperm injection, live birth delivery, idiopathic non-obstructive azoospermia, microdissection testicular sperm extraction

## Abstract

**Objective:**

To build a prediction model for live birth delivery per intracytoplasmic sperm injection (ICSI) in iNOA patients by obtaining sperm by microdissection testicular sperm extraction (mTESE).

**Methods:**

A retrospective cohort study of 377 couples with iNOA male partners treated with 519 mTESE-ICSI cycles was conducted from September 2013 to July 2021 at the Reproductive Medical Centre of Peking University Third Hospital. Following exclusions, 377 couples with iNOA male partners treated with 482 mTESE-ICSIs were included. A prediction model for live birth delivery per ICSI cycle was built by multivariable logistic regression and selected by 10-fold cross-validation. Discrimination was evaluated by c-statistics and calibration was evaluated by the calibration slope.

**Results:**

The live birth delivery rate per mTESE-ICSI cycle was 39.21% (189/482) in these couples. The model identified that the presence of motile sperm during mTESE, bigger testes, higher endometrial thickness on the day of human chorionic gonadotrophin (hCG) administration (ET-hCG), and higher quality embryos are associated with higher live birth delivery success rates. The results of the model were exported based on 10-fold cross-validation. In addition, the area under the mean ROC curve was 0.71 ± 0.05 after 10-fold cross-validation, indicating that the prediction model had certain prediction precision. A calibration plot with an estimated intercept of -1.653 (95% CI: -13.403 to 10.096) and a slope of 1.043 (95% CI: 0.777 to 1.308) indicated that the model was well-calibrated.

**Conclusion:**

Our prediction model will provide valuable information about the chances of live birth delivery in couples with iNOA male partners who have a plan for mTESE-ICSI treatment. Therefore, it can improve and personalize counseling for the medical treatment of these patients.

## 1 Introduction

According to the World Health Organization (WHO), approximately 15% of couples of childbearing age experience problems with fertility, and approximately half of these are caused by male factors. One of these factors, azoospermia, is characterized by the absence of sperm in two consecutive semen analyses according to WHO guidelines. Azoospermia is observed in 10-15% of infertile males and is classified as obstructive azoospermia and non-obstructive azoospermia (NOA). As the most severe infertility phenotype, NOA caused by testicular dysfunction comprises approximately 60% of azoospermia (1). Only approximately 28% of NOA patients can be diagnosed with clear causes, but nearly 70% of NOA patients were classified as idiopathic NOA (iNOA) for which the causes were unknown (2). Featured by higher success rate and lower testicular damage, the microdissection testicular sperm extraction (mTESE) is widely used for sperm retrieval in NOA patients, and testicular sperm retrieved by mTESE combined with intracytoplasmic sperm injection (ICSI) is currently the first-line treatment option for them to be able to father children (3, 4). Patients with different etiologies of NOA showed different sperm retrieval rates by 30%-75%, and the sperm retrieval rate of iNOA patients is the lowest one by 30% (5).

Couples with males who can obtain sperm by ejaculation semen could have their biological children through enough ICSI cycles. However, NOA patients just have a few chances, or even one, to get a few sperm by mTESE which means they just have a few chances, or even one, to accept ICSI treatments. Thus, ICSI outcomes using such precious sperm, especially live birth delivery rate, have drawn more and more attention from clinical experts in the assisted reproduction field.

The overall cumulative live birth rate of NOA couples was 46.82%, and NOA caused by Y chromosome azoospermia factor c (AZFc) microdeletions have the poorest embryological and clinical outcomes when compared with other etiologies (6). Among NOA patients with different etiologies, our data also demonstrated that the worst ICSI outcome in NOA patients with AZFc microdeletions compared with iNOA patients was caused by AZFc microdeletions (cumulative live birth delivery rate, 35.15%vs. 53.44%) (7, 8). Some studies showed that ICSI outcomes were not affected by male characteristics such as age, the state of sperm (fresh vs. frozen-thawed sperm), and hormone levels in NOA couples (9–11). Other literature exhibited that predictive factors of clinical pregnancy rate were the clinical type of azoospermia; male BMI; testicular volume; male levels of FSH, LH, and testosterone; female age; primary or secondary infertility (female); clinical type of female infertility; levels of AFC and AMH; number of oocytes; and number of oocytes used for ICSI (12–17). Other researchers have demonstrated that predictive factors of live birth were the type of azoospermia (OA vs. NOA), duration of infertility, first TESE-ICSI cycle, male levels of LH and testosterone, motility of spermatozoa for ICSI, and female age (14, 18).

Some papers have shown that good motility and morphology and a high quantity of sperm were associated with higher fertilization rates and greater odds of clinical pregnancy (6, 12, 18, 19). So far, there have been just two studies showing prediction models for ICSI outcomes in azoospermia patients. However the prognostic value of the models for live birth was limited because of a relatively low area under the receiver operating characteristic (0.62) in one of the studies and there was a model for clinical pregnancy in the other study (12, 18). Therefore, there is a shortage of studies that explore ICSI outcomes in iNOA patients after obtaining sperm by mTESE and that build a prediction model for live birth delivery.

As the largest population of NOA patients (~70%), iNOA patients have the lowest retrieval rate, so they have much fewer opportunities to obtain sperm and accept subsequent ICSI treatments. Thus, effectively utilizing these precious sperm in mTESE surgeries and having more chances to have their own biological offspring through ICSI is important to these patients. A prediction model for live birth delivery, which certainly is an overriding concern for both iNOA patients and doctors, will be valuable for these patients. These iNOA couples would benefit from the prediction model and could be well informed about their likelihood to have children before consenting to the mTESE treatment and subsequent ICSIs to help patients and doctors together make joint decisions and further select the optimal assisted reproductive techniques, treatments, and procedures.

## 2 Materials and methods

### 2.1 Subjects

**Figure 1:**
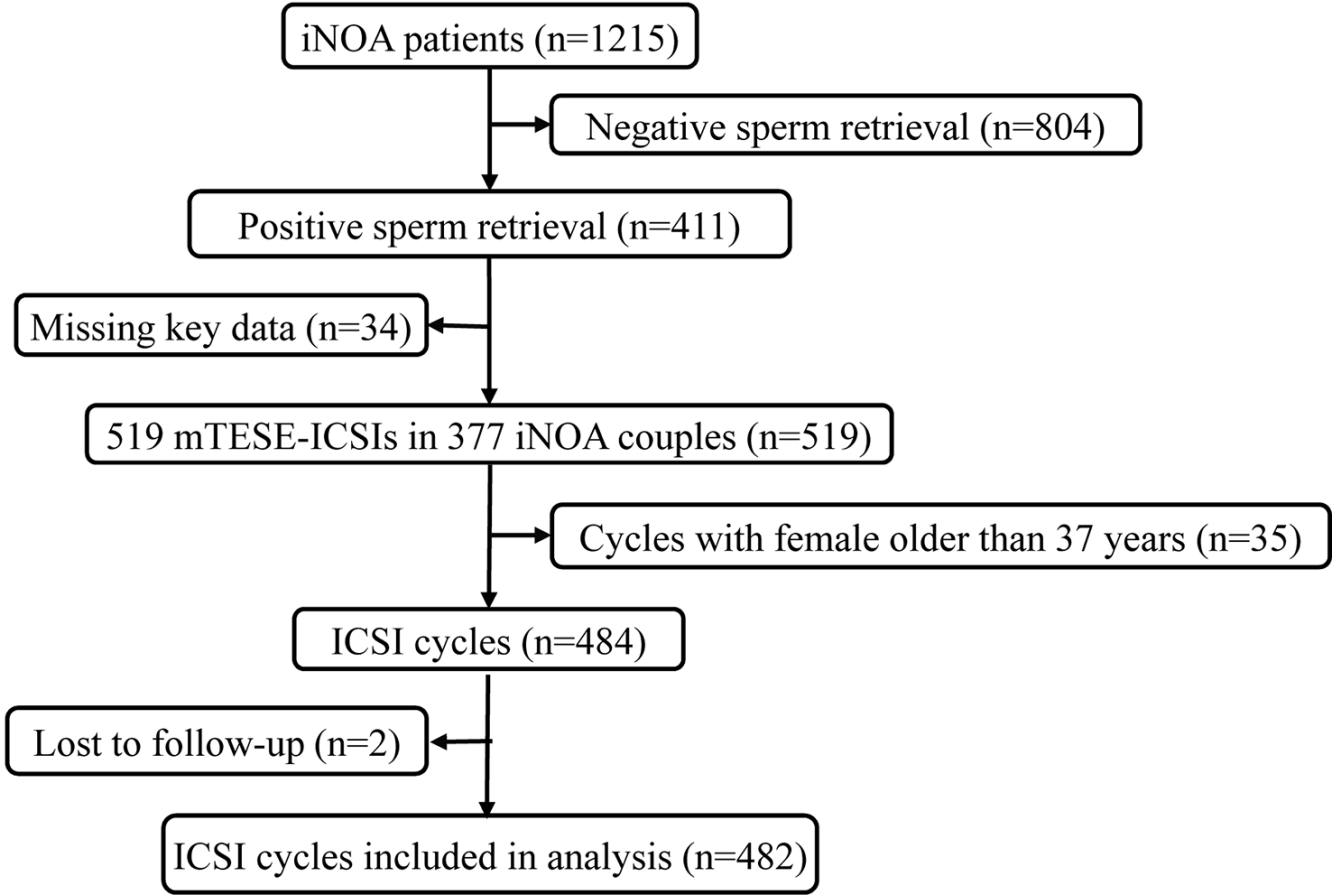
Flow chart of participants from 2013 to 2021 at the Reproductive Medical Centre of Peking University Third Hospital. A total of 1,215 participants were included. After excluding participants with negative sperm retrieval during mTESE surgeries (n=804) and those lacking key data (n=34), 377 iNOA patients were included. After further excluding ICSI cycles with females older than 37 years (n=35) and those lost to follow-up (n=2), 482 ICSI cycles were included in our final analysis. iNOA, idiopathic non-obstructive azoospermia; ICSI, intracytoplasmic sperm injection.

This retrospective study was conducted from September 2013 to July 2021 on 1,215 patients with iNOA undergoing mTESE and 377 couples with iNOA male partners who previously had not received treatment of assisted reproductive technology and who were treated with 519 mTESE-ICSI cycles at the Reproductive Medical Centre of Peking University Third Hospital. Before mTESE, each male underwent a complete andrological evaluation for iNOA. The etiology of iNOA was determined by semen analysis when there are no spermatozoa in at least two consecutive semen analyses according to the WHO criteria, testicular volume evaluation, FSH concentration, and transrectal ultrasonographic examination of the prostate and seminal vesicles after excluding evidence of obstruction and other NOA with clear causes (20, 21). To eliminate the effect of female age on the ICSI outcomes, couples in which the female partners were older than 37 years were excluded, which means all female partners were young women (22–24). Additionally, couples using cryopreserved oocytes and donor semen were also excluded. Subsequently, there were 482 ICSI cycles left after excluding females older than 37 years and cycles with frozen-thawed oocytes (**Figure 1**). Finally, we retrospectively collated the records of these couples after mTESE-ICSI by telephone follow-up.

### 2.2 Ethical approval

The current study involving human participants was reviewed and approved by the Ethics Committee of Peking University Third Hospital (Beijing, China, state reference number 2023-198-02). All patients involved in this study provided their written informed consent to participate in this study.

### 2.3 Preparation of sperm for ICSI

All the mTESE procedures were performed under general or spinal anesthesia in accordance with previously published guidelines (25), with some modifications. The skin of the testis was incised along the scrotal midline and through the tunica vaginalis using a scalpel. The tunica albuginea was then incised with a scalpel near its midportion under an operating microscope (OPMI Vario/S88 System, Karl Zeiss, Germany) to optimize visualization of the testicular parenchyma without affecting the testicular blood supply. Examination of the testicular parenchyma was conducted at×12–24 magnification under the operating microscope. The thick and opaque seminiferous tubules harvested in mTESE surgery were picked out and minced with a pair of 1-mL sterile syringe needles into a homogeneous suspension in a dish containing G-MOPS-plus medium (Vitrolife, Vastra Frolunda, Sweden). Next, we used an inverted microscope (Nikon TE2000-U, Japan) at ×200 magnification to observe the sperm. When the amount of sperm was less than 50, the sperm were counted out and recorded. Then, the total sperm amount was defined as a low sperm count when the number of sperm observed under the microscope was fewer than or equal to 20. Once sperm were identified in a suspension, we centrifuged the suspension for 10 minutes at 450×g. The resulting pellet was then pipetted into droplets and covered with mineral oil in a dish for use in subsequent ICSIs. When the sperm acquisition was not synchronized with the partner’s ovarian stimulation on the day of the mTESE surgery, the cell suspension containing the sperm was mixed with a sperm freeze solution (Vitrolife) at a ratio of 1:1 in a 2-mL straw. The straw was left at room temperature for 10 minutes, placed in a liquid nitrogen bath for 30 minutes, and then stored in a liquid nitrogen canister. When required, the frozen mTESE sperm were placed in a 37°C incubator for 15 minutes, mixed with 2 mL of washing medium, and centrifuged at 450×g for 10 min. Subsequently, the frozen-thawed micro-TESE sperm was used for the ICSI, as described above.

### 2.4 Ovarian stimulation, oocyte retrieval, and ICSI

A gonadotropin-releasing hormone agonist or antagonist was used for ovarian hyperstimulation in the female partner (26). Measurement of the follicles by transvaginal ultrasonography and serum estradiol levels were conducted to detect follicular development. When the concentration of estradiol was >500 pg/mL simultaneously accompanied by the presence of≥1 follicle of 18 mm, we administered the patient 10,000 units of urinary human chorionic gonadotropin (Serono, Aubonne, Switzerland). Oocyte retrieval was performed with the help of a transvaginal ultrasound 36–38 hours after the administration of human chorionic gonadotropin. The cumulus cells were separated from the oocyte by pipetting and exposure to hyaluronidase (Type VIII; Sigma Chemical Company, St. Louis, MO, USA) 2 hours after retrieval. After the selection of the appropriate sperm, an ICSI was performed by pipette injection under the control of a micromanipulator on the day of oocyte retrieval (day 0) using previous recommendations (PALERMO et al., 1992). Luteal support was provided by 60 mg of progesterone (Xianju Pharmacy, Zhejiang, People’s Republic of China) on the next day after oocyte retrieval (27).

### 2.5 Embryo transfer

Injected oocytes were cultured in appropriate media at 37°C in an incubator with an atmosphere of N2/CO2/O2 (90:5:5, v/v). The presence of 2 pronuclei (2PN) in an embryo indicated the successful creation of a zygote 17–19 hours after ICSI, and the zygote was cultured in 25 mL of preequilibrated cleavage medium (G1, Global HTF, and Quinn’s advantage cleavage medium) in an incubator, as mentioned above, until uterine transfer. Embryos were scored in accordance with the Society for ART scoring system (28) by checking cell number, fragmentation, and cell symmetry on either day 3 or day 5 after ICSI; the highest quality embryos were then transferred into the uterus. The remaining embryos were cultured to the blastocyst stage for cryopreservation or were frozen directly. If ovarian hyperstimulation syndrome developed, or there were other reasons why embryos could not be transferred, embryos were also cultured to the blastocyst stage for cryopreservation or frozen directly for future frozen-thawed cycle procedures to be performed in accordance with previously published guidelines (29).

### 2.6 Model development

The endpoint of this study was the first live birth delivery which was defined as the birth of a single baby or multiple after a complete cycle of ICSI (30). Live birth delivery is defined as at least one baby surviving for more than 1 month. A complete cycle is defined as the results from all fresh and frozen-thawed embryo transfers or the first live birth delivery resulting from a single administration of drugs to reinduce superovulation. Our current aim was to construct a prediction model for live birth delivery at the cycle level, therefore each cycle was considered as a separate unit of analysis.

Univariable analysis was used to identify potential predictors for live birth delivery. Subsequently, based on univariable analysis (p<0.05) and knowledge from the existing literature (6, 12–18), the candidate prognostic parameters were the type of female infertility (normal, tubal factors, endometriosis, polycystic ovary syndrome, congenital uterine anomaly, and acquired uterine anomaly), age (years, male and female), body mass index (BMI, kg/m^2^, male and female), follicle-stimulating hormone (FSH, IU/l, male and female), luteinizing hormone (LH, IU/l, male and female), testosterone (nmol/l, male), estradiol (pmol/l, female), progesterone (P, nmol/l, female), total volume of testes (ml), motility of sperm during mTESE (yes/no), number of metaphase II oocytes (n), endometrial thickness on the day of human chorionic gonadotrophin (ET-hCG) administration (ET-hCG, mm), and high-quality embryos (n).

Before constructing the model, a collinearity test was used to evaluate the collinearity of variables to avoid the inclusion of redundant variables in the model. Forward and backward stepwise selection procedures based on the log-likelihood ratio were used to select the predictors in the model, and the selection criteria was a p-value of 0.20 to assure more liberal inclusion of potential predictors (31). Multivariable logistic regression analysis with an enter procedure was used to develop a final well-fitting prediction model for its maximum accuracy in calculating the probability of live birth delivery. Finally, an optimal multiple regression model including reliable potential predictors was achieved with Wald p < 0.05 for entry and p> 0.1 for removal.

### 2.7 Missing data

Variables with missing data, including FSH and BMI, were normally distributed data, and regression imputation is more applicable to handling missing data subject to normal distribution. Thus, it was used to deal with those predictors with missing information, and valid statistical inference and point estimation could be performed based on this complete dataset.

### 2.8 Model validation

To reduce the overfitting of the model, internal validation was carried out by k-fold cross-validation to quantitatively assess the accuracy of this model. This method involves splitting the dataset into N equal size folds. N-1 folds were used as training data to build a model and the remaining one fold was used as test data to estimate the performance of the model. This process was repeated N times, with different training and test data used each time. Finally, the average of N results was used to estimate the discrimination of the model. Furthermore, the entire dataset could be used for model development without being wasted by this method, so it is a generally reliable and commonly used approach to evaluate modeling methodologies (32, 33). Normally, 10-fold cross-validation performs well and provides a good tradeoff between evaluation variance and estimation variance. Thus, 10-fold cross-validation was performed to test the predicted live birth delivery. Then, multivariate logistic regression analysis was performed in the 10 models and the average model from the 10 models was generated. We calculated the predicted live birth delivery based on the model and compared it with the actual live birth delivery to validate the discrimination and calibration abilities of the model.

### 2.9 Performance of the model

Discrimination is defined as a model’s ability to correctly distinguish non-events and events can be quantified by calculating the area under the receiver operating characteristic (ROC) curve developed for the model (AUC). Here, discrimination refers to the ability of the model to find out whether a couple could have a live birth delivery. The performance of the developed models was assessed using ROC with 10-fold cross-validation and 10 ROC curve with 10-fold cross-validation was developed through Python and its commands. Calibration measures how closely the predicted probabilities agree numerically with the actual outcomes, so the calibration of the model was evaluated with the Hosmer and Lemeshow test for goodness-of-fit. In order to make the test results more visible and clear, we used calibration plots to verify the consistency between the prediction probability and the actual outcome. Next, a nomogram was developed through R and its commands to calculate the total score for predicting live birth delivery, and this visible clinical decision tool in the form of a score chart could be used in clinical practice.

### 2.10 Statistical analysis

All statistical analyses were performed with SPSS 26.0 (SPSS Inc., Chicago, IL), R version 4.0.2 (The R Foundation for Statistical Computing, Vienna, Austria), and Python version 3.6 (Python Software Foundation, Delaware, United States of America).

## 3 Results

### 3.1 Follow-up results and clinical characteristics of the participants

**Figure 1** shows a schematic overview of the participants. A total of 1,215 iNOA patients underwent mTESE, and sperm were retrieved in 411 patients (sperm retrieval rate, SRR=33.83%). ICSIs were performed in 377 iNOA couples after excluding cases without thawing sperm or key data such as volume of testes, male age, male FSH, female age, and ET-hCG. Finally, a total of 482 mTESE-ICSI cycles were included in this study after following the exclusion criteria. The overall basic clinical information of the couples is exhibited in **Table 1**, and the live birth delivery rate per mTESE-ICSI cycle was 39.21% (189/482) in iNOA couples in the current study.

**Table 1:** Baseline characteristics of the couples with iNOA male partners.

	Total cycles	Live birth Delivery	No Live birth Delivery
Number of complete cycles (n)	482	189	293
Duration of infertility (years), median (IQR)	3 (2, 5)	3 (2, 5)	3 (2, 5)
Male clinical characteristics			
Age (years), (mean ± SD)	31.34 ± 4.319	31.32 ± 4.243	31.35 ± 4.375
BMI (kg/m^2^), (mean ± SD)	25.52 ± 3.990	25.65 ± 3.814	25.43 ± 4.103
Total volume of testes (ml), median (IQR)	12 (10, 20)	12 (10, 20)	12 (8, 18)
FSH (mIU/ml), median (IQR)	20.70 (13.45, 27.13)	21.3 (14.35, 28.30)	20.3 (12.65, 26.90)
LH (mIU/ml), median (IQR)	8.46 (5.60, 11.30)	8.41 (5.61, 11.20)	8.54 (5.575, 11.40)
T (nmol/l), median (IQR)	8.74 (6.03, 11.50)	8.84 (6.04, 11.50)	8.74 (6.03, 11.70)
PRL (ng/ml), median (IQR)	10.6 (7.14, 10.6)	10.60 (7.44, 11.23)	10.6 (7.04, 11.16)
Motile sperm during mTESE, n (%)			
Yes	399 (82.78)	181 (95.77)	218 (74.40)
No	83 (17.22)	8 (4.23)	75 (25.60)
Female parameters per cycle, n (%)			
Endometriosis	3 (0.62)	1 (0.53)	2 (0.68)
Polycystic ovary syndrome	33 (6.85)	12 (6.35)	21 (7.17)
Congenital uterine anomaly	6 (1.24)	2 (1.06)	4 (1.37)
Acquired uterine anomaly	20 (4.15)	3 (1.59)	17 (5.80)
Female clinical characteristics			
Age (years), (mean ± SD)	29.85 ± 3.313	29.62 ± 3.122	29.99 ± 3.428
BMI (kg/m^2^), (mean ± SD)	22.53 ± 4.055	22.33 ± 4.559	22.67 ± 3.695
No. of AFC, median (IQR)	12 (9, 15)	12 (9, 15)	11 (8, 14)
ET-hCG(mm), median (IQR)	11 (10, 12)	11 (10, 12)	11 (10, 12)
FSH (mIU/ml), median (IQR)	5.30 (2.95, 6.69)	5.30 (2.92, 6.67)	5.30 (3.00, 6.69)
LH (mIU/ml), median (IQR)	2.32 (1.13, 4.14)	2.79 (1.20, 4.22)	2.22 (1.07, 4.02)
E_2_ (pmol/l), median (IQR)	139 (109, 171)	141 (108, 173.50)	139 (109, 169.50)
P (nmol/l), median (IQR)	1.10 (0.80, 1.41)	1.11 (0.79, 1.41)	1.08 (0.80, 1.42)
Laboratory data per cycle, mean (IQR)			
No. of metaphase II oocytes	10 (7, 15)	12 (8, 16)	10 (6, 14)
No. of high-quality embryos	4 (1, 6)	5 (3, 8)	3 (1, 5)

Results are reported as mean ± SD and median and IQR. ICSI, intracytoplasmic sperm injection; SD, standard deviation; IQR, interquartile range; BMI, body mass index; FSH, follicle-stimulating hormone; LH, luteinizing hormone; T, testosterone; PRL, pituitary prolactin; Acquired uterine anomaly, uterus myomatosus, endometrial polyp or adenomyosis; E_2_, estradiol; P, progesterone; No., number; AFC, antral follicle count; ET-hCG, Endometrial thickness on the day of human chorionic gonadotrophin (hCG) administration.

### 3.2 Model development and internal validation

In total, 6 (1.58%) and 6 (1.58%) couples were missing female BMI and FSH, respectively, and the other variables did not have any missing data. We used the regression imputation method for handling missing data. The final multivariable logistic regression model for live birth delivery with all candidate predictors demonstrated that the four predictors that met the predefined selection criteria were motility of sperm during mTESE, testicular volume, ET-hCG, and the number of high-quality embryos. Among these variables, the motility of sperm during mTESE was a dichotomous variable and the remaining three were continuous variables (**Table 2**).

**Table 2:** Multivariable logistic regression model for live birth delivery with mTESE-ICSI.

	β	*P* value	OR	95% CI
Motile sperm during mTESE (yes/no)	-1.499	<0.001	0.223	0.099-0.505
Total volume of testes (ml)	0.039	0.021	1.040	1.006-1.075
ET-hCG (mm)	0.144	0.044	1.155	1.004-1.329
No. of high-quality embryos	0.148	<0.001	1.160	1.070-1.258
Constant	-4.860			

ICSI, intracytoplasmic sperm injection; mTESE, microdissection testicular sperm extraction; No., number; ET-hCG, endometrial thickness on the day of hCG administration; β, regression coefficient; CI, confidence interval.

Then, the final predictive equation to estimate the probability of live birth delivery based on the regression coefficient as follows: P(success)= 100%*1/{1+exp[-(-4.860-1.499*motile sperm during mTESE+ 0.039*total volume of testes+0.144* ET-hCG+0.148*No. of high quality embryos]}. The predictors that appeared to be significantly positively correlated with live birth delivery were testicular volume, ET-hCG, and the number of high-quality embryos. Bigger testes were associated with higher odds of live birth delivery (OR:1.040, 95%CI: 1.006 to 1.075). More high-quality embryos at the ICSI cycle also enhanced the chance of success (OR:1.160, 95%CI: 1.070 to 1.258), and increasing ET-hCG increased the chance of having a live birth delivery as well (OR:1.155, 9%CI: 1.004 to 1.329). Nevertheless, the absence of motile sperm during mTESE appeared to be significantly negatively correlated with live birth delivery, and the lack of motile sperm during mTESE reduced the odds of live birth delivery (OR: 0.223, 95% CI: 0.099 to 0.505) (**Table 2**).

**Figure 2:**
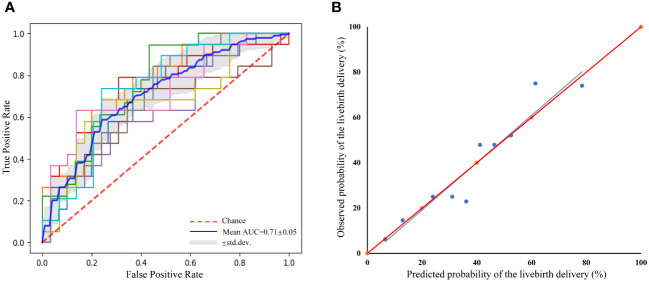
Receiver operating characteristic curve (ROC) of the prediction model for the prediction of live birth delivery based on 10-fold cross-validation and a calibration plot for the prediction of live birth delivery. **(A)** ROC curve. mean area under the curve=0.71 ± 0.05, indicating reasonable discriminative performance, **(B)** Calibration plot. R^2 =^ 0.911, y= -1.653 + 1.043x; Slope=1.043(95%CI 0.777–1.308); Intercept=-1.653 (95%CI -13.403–10.096).

Our calibration plot showed the model was well-calibrated and had good predictive accuracy between the actual probability and predicted probability with the estimated intercept of -1.653 (95% CI: -13.403 to 10.096) and the slope of 1.043 (95% CI: 0.777 to 1.308) (**Figure 2**). The intercept approached zero and the slope unity. The predicted possibility of a live birth delivery ranged from 6.54% to 78.45% with a mean of 39.05%. Among the ICSI cycles, 25% of them had a probability of less than 20.83% and 25% of them had a probability exceeding 57.56%.

### 3.3 Performance of the model

The mean AUC was 0.71 ± 0.05, indicating that the prediction model for live birth delivery in iNOA couples had certain prediction precision (**Figure 2**). Furthermore, the goodness-of-fit test (Hosmer-Lemeshow) demonstrated no significant miscalibration (P=0.274), indicating a good overall performance of the model.

**Figure 3:**
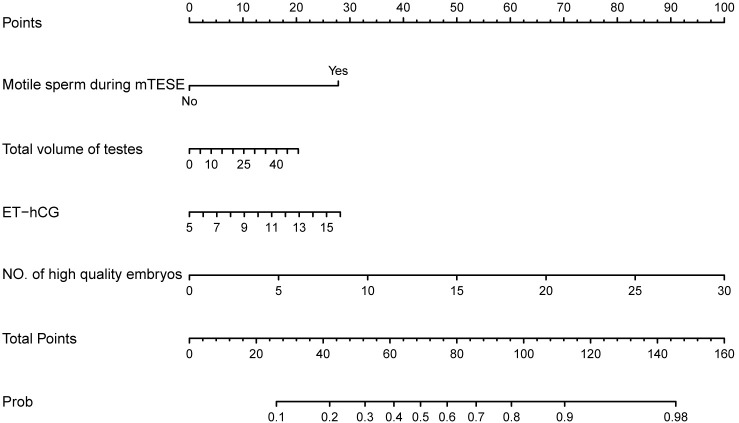
Nomogram chart with four variables to predict live birth delivery in couples with iNOA male partners after mTESE-ICSI treatments. ICSI, intracytoplasmic sperm injection; mTESE, microdissection testicular sperm extraction; ET-hCG, endometrial thickness on the day of hCG administration; NO., number; Prob, Probability of live birth delivery.

Based on the final regression analysis, a more visual representation of this score chart was displayed in the nomogram, including the four significant factors for predicting live birth delivery (**Figure 3**). The value of each variable was given a score on the points scale axis by drawing a straight line from the factor scale to the points scale. The total points can be calculated by adding each single score, and the probability of live birth delivery can be estimated by projecting the total points to the ‘probability of live birth delivery’ scale.

## 4 Discussion

As the standard therapy scheme for NOA patients, an ICSI following mTESE is now widely and regularly used for the treatment of their infertility. Compared with males obtaining sperm through ejaculated semen, NOA patients had to accept invasive surgery, mTESE, for retrieving testicular sperm. Worse still, NOA patients have just a few, or even one, opportunities for mTESE surgeries, so people are more and more concerned about ICSI outcomes using rare and precious testicular sperm. Templeton et al. (1996) reported that female age was inversely correlated with the live birth rate (34). Besides female age, other factors, including duration of subfertility, primary or secondary infertility, and percentage of motile sperm, have been advocated as possible prognostic indicators for successful live births (35). Meijerink et al. showed that a successful live birth was associated with lower female age, first TESE-ICSI cycle, lower male LH, higher male testosterone, the use of motile spermatozoa for ICSI, and having obstructive azoospermia in couples undergoing ICSI using testicular sperm (18). Recently, our data and another two studies demonstrated that the type of azoospermia was also related to the live birth rate in couples with NOA male partners (5, 6, 8).

Thus, the prediction of ICSI outcomes after mTESE has attracted the attention of clinical research in recent years, but there were two articles that showed prediction models for live birth or pregnancy in azoospermia patients (12, 18). One article, displaying a prediction model for clinical pregnancy, exhibited that the clinical type of azoospermia, testicular size, male FSH, male LH, male testosterone, female age, female antral follicle count (AFC), and female anti-Mullerian hormone (AMH) were used as predictors in couples with azoospermia male partners (12). Another study showing a prediction model for live birth rate in ICSI using testicular extracted sperm indicated that female age, a first or subsequent started TESE-ICSI cycle, male LH, male testosterone, motility of the spermatozoa during the ICSI procedurem and clinical type of azoospermia were identified as relevant and independent parameters for live birth (18).

In this retrospective study, which is the largest sample size study about ICSI outcomes of couples with iNOA male partners including 482 ICSI cycles at present, we systematically and comprehensively analyzed, for the first time, influencing factors of live birth delivery which is the most important endpoint of assisted reproductive technology for these couples and what they really want to know the most before accepting mTESE-ICSI treatments. Furthermore, we developed and validated a reliable and valuable model for live birth delivery with obvious clinical application value for iNOA couples. Moreover, based on the model development and validation by multivariable logistic regression and calibration analyses, the model had good calibration and moderate discrimination, meaning it could be used in clinical practice.

Our prospective follow-up of the outcomes of 482 ICSIs in iNOA patients demonstrated a live birth delivery rate per mTESE-ICSI cycle of 39.21%, which is similar to a previously reported live birth outcome of 40.6% (5). Female partners were all young women in our study, so female age was not included as a predictor in our model unlike other studies (23, 24). The principal strength of our study is its novelty and it has important implications for clinical practice based on the available evidence. We found similar predictors, such as the number of high-quality embryos and ET-hCG, as previous studies on IVF/ICSI (12, 14, 17). There was just one prediction model for live births with unsatisfactory discrimination (AUC=0.62) and one prediction model for clinical pregnancy in azoospermia patients (12, 18). However, these models are not applicable to iNOA couples carrying specific predictors. Moreover, our model is specifically designed for iNOA patients obtaining sperm at mTESE who have a plan for starting ICSI treatment. Thus, our model addresses a key unmet need in terms of informing these couples of their probability of live birth delivery since sperm retrieved at mTESE is more precious for iNOA patients who need to bear the extra expense, trauma, and stress caused by surgery than those who have sperm extracted from semen or testicular sperm aspiration, especially as some of them have only one chance of mTESE surgery. Next, the model developed in the study containing the latest data expands on the current literature and extends the use of clinical prediction models in mTESE-ICSI treatment.

For iNOA couples, we found that larger testicular volume is a predictive value for live birth delivery based on our model. For NOA patients, changes of some seminiferous tubules in the structure and function resulting in local spermatogenic dysfunction allow the preservation of tubule foci with normal residual activity. The positive correlation between testicular volume and sperm retrieval rate in azoospermia patients (36) suggests that there are more residual seminiferous tubule foci with normal spermatogenesis and a lower level of degree of spermatogenic dysfunction. A recent study showed that azoospermia patients with larger testicular volumes have a higher pregnancy rate (12), which indicates a positive correlation between this predictor and live birth delivery.

In our study, the number of high-quality embryos and ET-hCG were significantly positively associated with the chance of success. Previous studies reporting the association between an increasing number of high-quality embryos and significantly higher chances of pregnancy after IVF treatment indicate theoretically the positive correlation between the number of high-quality embryos and live birth delivery, which is in agreement with our findings (37). Studies have reported that endometrial thickness of more than 7–10 mm and 10-12 mm (in frozen-thaw and fresh embryo transfer cycles) and less than 6 mm were associated with increasing and reducing live birth rate respectively (38, 39),, which is consistent with our model.

The model also identified the motility of sperm during mTESE as another predictor in addition to the above predictors, and the absence of motile sperm during mTESE was an adverse factor for the chance of having a live birth delivery. In iNOA patients without any motile sperm during mTESE, spermatogenic dysfunction is so severe that more retrieved sperm are teratozoospermia, reflecting the lack of integrity and functional competence. Some studies reported that the motility of sperm from NOA patients was significantly associated with poorer clinical outcomes (6, 19), and sperm from NOA patients showing aneuploidy, mosaicism, and DNA damage contributed to poorer clinical outcomes (40). Therefore, for iNOA patients without any motile sperm during mTESE, it may be that more sperm with aneuploidy or mosaicism and an increased degree of DNA damage is one of the causes of a lower likelihood of achieving live birth delivery.

The applicability of our findings in different clinical settings may have certain limitations. First, all female partners included in the study were young fertile women (<38 years old), so our model cannot apply to those couples with female partners older than 37 years. Second, we had a lack of data on the morphology and motility of sperm used for ICSI due to the limited availability of covariate information in our dataset. Several studies have reported that low-mobility sperm and more abnormal sperm used for ICSI could result in poor clinical outcomes (6, 12, 18, 19). Finally, another limitation of our prediction model is that we did not evaluate its external validity and clinical impact in another population from another center because our sample size was already the largest so far.

Evidence-based counseling, followed by shared decision-making of couples undergoing mTESE-ICSI, is very important since doctors working with these couples in the process of assessing the chances of live birth deliveries can then meet the latter’s needs and wishes. Joint decisions from doctors and couples make the patients feel strongly involved so that couples that experience unsatisfactory ICSI outcomes seldom experience decisional conflicts and regret. 

## 5 Conclusion

In conclusion, in this study, we developed and validated a novel clinical prediction model that could be used to predict individualized chances of live birth delivery in iNOA couples starting an ICSI cycle following sperm retrieval at mTESE. Our model provides valuable information for a better understanding of the relationship of several main parameters related to mTESE-ICSI cycles for these couples and may help doctors in counseling them regarding expected live birth delivery. Finally, further studies that include iNOA patients from another IVF center are needed to confirm the prediction ability of the model.

## Data availability statement

The raw data supporting the conclusions of this article will be made available by the authors, without undue reservation.

## Ethics statement

The studies involving humans were approved by Ethics Committee of Peking University Third Hospital. The studies were conducted in accordance with the local legislation and institutional requirements. The participants provided their written informed consent to participate in this study.

## Author contributions

LZ designed the study and drafted the main manuscript. YW and LL collected the follow-up data. W-hT collected the patients’ data. LZ and YW performed the statistical analysis. LZ and XZ analyzed the data. JQ, RL, and PL revised the manuscript. All authors contributed to the article and approved the submitted version.
